# Supplementary figures and images for: SPARE-Tau: A flortaucipir machine-learning derived early predictor of cognitive decline
Source: PLoS One. 2022 Nov 3;17(11):e0276392. doi: 10.1371/journal.pone.0276392 (PMC9632811; doi:10.1371/journal.pone.0276392)

Supplementary Figure 1. Brain regions contributing to SPARE-Tau index.


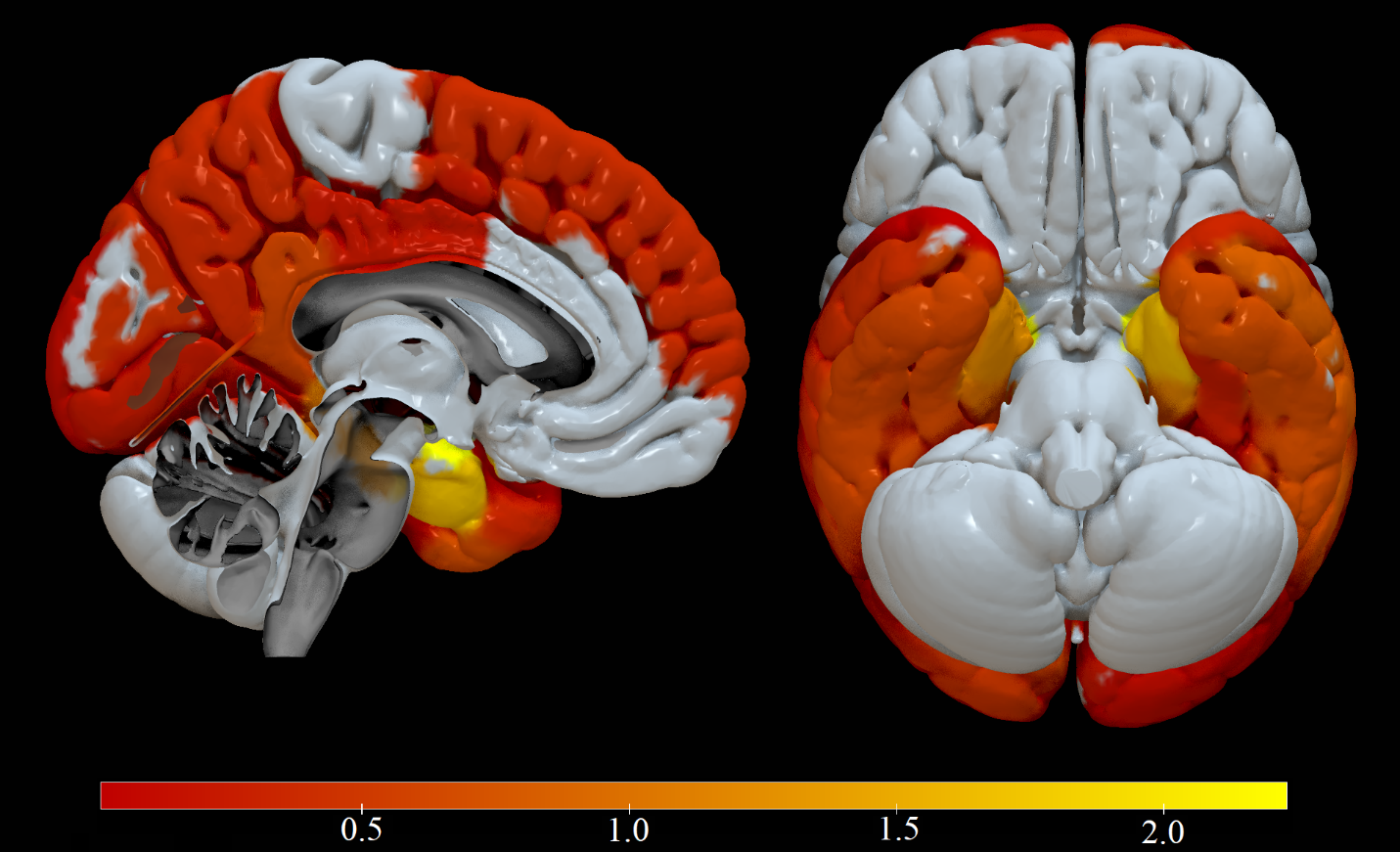

Supplement: S1 Fig — (DOCX) [file pone.0276392.s001.docx]
