# Supplementary material for: SPARE-Tau: A flortaucipir machine-learning derived early predictor of cognitive decline
Source: PLoS One. 2022 Nov 3;17(11):e0276392. doi: 10.1371/journal.pone.0276392 (PMC9632811; doi:10.1371/journal.pone.0276392)
Supplement: S1 Table — (DOCX) [file pone.0276392.s002.docx]

**Supplementary Table 1.** Clinical and biomarker characteristics stratified by clinical diagnosis at baseline in the training participant group (top: CU with normal Aβ biomarker values and cognitively impaired with pathological Aβ biomarker values) and the early preclinical validation group (CU with pathological Aβ biomarker values).

|  | Participants in Training Group | | | | Additional Participants | | | |
| --- | --- | --- | --- | --- | --- | --- | --- | --- |
| Variable | CU Aβ- (n=218) | MCI Aβ+ (n=93) | Dementia Aβ+ (n=56) | p-value | CU Aβ+ (n=126) | MCI Aβ- (n=89) | Dementia Aβ- (n=5) | p-value |
| Age at baseline (years) | 70.9  [(67.2)-(76.4)] | 75.7  [(69.7)-(80.1)] | 75.69 [(70.3)-(82.4)] | 0.0001 | 74.3 [(69.4)-(78.6)] | 72.5 [(68.3)-(81.5)] | 78.0 [(75.3)-(78)] | 0.79 |
| Sex (% Male) | 37.3% | 54.8% | 57.4% | 0.002 | 42.1% | 59.6% | 60% | 0.03 |
| APOE ε4 Presence (%) | 27.4% | 59.3% | 64.8% | 0.0001 | 50.4% | 13.4% | 0% | 0.0001 |
| ADAS-Cog 13 | 7.3  [(5)-(10.3)] | 16.7  [(12)-(22)] | 30.5  [(25)-(38.3)] | <0.0001 | 7.16 [(5)-(11.58)] | 12 [(9)-(15.3)] | 24 [(22.67)-(25.67)] | <0.0001 |
| Florbetapir: Composite score | 0.7  [(0.67)-(0.73)] | 0.94 [(0.84)-(1.0)] | 1  [(0.95)-(1.1)] | <0.0001 | 0.84 [(0.79)-(0.96)] | 0.7 [(0.66)-(0.71)] | 0.67 [(0.64)-(0.69)] | <0.0001 |
| CSF P-Tau () | 18.3  [(15.0)-(22.2)] | 25.1  [(19.0)-(42.6)] | 33.5  [(26.1)-(44.1)] | <0.0001 | 24.22  [(16.28)-(33.46)] | 20.2  [(17.3)-(26)] | 13.3  [(13)-(13.6)] | 0.039 |
| Flortaucipir Meta-temporal ROI | 1.49  [(1.4)-(1.6)] | 1.8  [(1.6)-(2.1)] | 2.29  [(1.8)-(3.1)] | <0.0001 | 1.56 [(1.48)-(1.69)] | 1.52  [(1.43)-(1.63)] | 1.61 [(1.6)-(1.61)] | 0.022 |
| Flortaucipir: Global Tau Score | 1.49 [(1.4)-(1.6)] | 1.85 [(1.6)-(2.1)] | 2.27  [(1.8)-(2.9)] | <0.0001 | 1.58 [(1.48)-(1.74)] | 1.52  [(1.46)-(1.69)] | 1.58  [(1.57)-(1.7)] | 0.17 |
| Flortaucipir: SPARE Tau | -1.1  [(-1.4)-(-0.8)] | 0.86  [(-0.61)-(2.8)] | 4.46  [(1.3)-(8.2)] | <0.0001 | -0.68  [(-1.17)-(0.15)] | -1  [(-1.3)-(-0.33)] | -0.37  [(-0.37)-(-0.37)] | 0.032 |
| MRI: SPARE AD | -1.8  [(-2.5)-(-1.1)] | -0.47  [(-1.4)-(0.5)] | 1.3  [(-0.17)-(3.1)] | <0.0001 | -1.45  [(-2.1)-(-0.66)] | -1.24  [(-2.12)-(-0.18)] | -0.05  [(-2.2)-(0.11)] | 0.52 |
| Brain Age Gap | -1.14 [(-3.86)-(2.11)] | 1.58 [(-1.26)-(4.66)] | 3.27 [(0.52)-(8.03)] | <0.0001 | -0.5  [(-3.71)-(2.58)] | 0.1 [  (-3.99)-(4.47)] | 6.64  [(5.25)-(8.74)] | 0.003 |
