# Supplementary material for: SPARE-Tau: A flortaucipir machine-learning derived early predictor of cognitive decline
Source: PLoS One. 2022 Nov 3;17(11):e0276392. doi: 10.1371/journal.pone.0276392 (PMC9632811; doi:10.1371/journal.pone.0276392)
Supplement: S2 Table — Table presents mean R2 and 95% CI of the 1000 bootstrapped models. (DOCX) [file pone.0276392.s003.docx]

**Supplementary Table 2**. **Cross-sectional prediction of ADAS-Cog13 scores using multivariate adaptive regression splines models.** Table presents mean R^2^ and 95% CI of the 1000 bootstrapped models.

| Biomarker | Mean R^2^ (95% CI) | |
| --- | --- | --- |
|  | Aβ+ | Aβ- |
| SPARE-Tau | 0.58 [(0.44)-(0.73)] | 0.15 [(-0.09)-(0.39)] |
| Average Tau | 0.55 [(0.39)-(0.7)] | 0.06 [(-0.1)-(0.21)] |
| Meta-Temporal ROI | 0.49 [(0.33)-(0.66)] | 0.09 [(-0.1)-(0.29)] |
| CSF p-Tau | 0.11 [(-0.01)-(0.22)] | 0.02 [(-0.07)-(0.11)] |
| Florbetapir Composite | 0.29 [(0.08)-(0.5)] | 0.14 [(-0.12)-(0.41)] |
| SPARE-AD | 0.5 [(0.32)-(0.67)] | 0.09 [(-0.07)-(0.25)] |
| Global 1 | 0.73 [(0.6)-(0.85)] | 0.25 [(-0.04)-(0.55)] |
| Global 2 | 0.74 [(0.62)-(0.85)] | 0.39 [(0.12)-(0.67)] |
