# Supplementary material for: SPARE-Tau: A flortaucipir machine-learning derived early predictor of cognitive decline
Source: PLoS One. 2022 Nov 3;17(11):e0276392. doi: 10.1371/journal.pone.0276392 (PMC9632811; doi:10.1371/journal.pone.0276392)
Supplement: S3 Table — Paired t-test analysis. Bonferroni-Holm multiple comparison correction. (DOCX) [file pone.0276392.s004.docx]

**Supplementary Table 3. Comparison of baseline fit of ADAS-Cog13 scores (R^2^ values) for each pair of multivariate adaptive regression splines models.** Paired t-test analysis. Bonferroni-Holm multiple comparison correction.

| Biomarker 1 | Biomarker 2 | Aβ+ | | | | Aβ- | | | |
| --- | --- | --- | --- | --- | --- | --- | --- | --- | --- |
|  |  | t | Adjusted p-value | Difference | 95% CI | t | Adjusted p-value | Difference | 95% CI |
| SPARE-Tau | Average Braak | 23.3 | <0.0001 | 0.0358 | [(0.033 - 0.039)] | 29.8 | <0.0001 | 0.0931 | [(0.0869)-( 0.0992)] |
| SPARE-Tau | Meta-Temporal ROI | 49.5 | <0.0001 | 0.0918 | [(0.09)-(0.10)] | 16.8 | <0.0001 | 0.0593 | [(0.05243)-( 0.0663)] |
| SPARE-Tau | CSF p-Tau | 163 | <0.0001 | 0.4793 | [(0.47-0.49)] | 31.2 | <0.0001 | 0.1286 | [(0.121)-( 0.137)] |
| SPARE-Tau | SPARE-AD | 35.7 | <0.0001 | 0.0879 | [(0.08-0.09)] | 14.9 | <0.0001 | 0.0617 | [(0.0536)-( 0.0698)] |
| SPARE-Tau | Florbetapir Composite | 90.3 | <0.0001 | 0.2898 | [(0.28- 0.30)] | 1.6 | 0.12 | 0.0091 | [(-0.00218)-( 0.0205)] |
| SPARE-Tau | Global 1 | -72.4 | <0.0001 | -0.1421 | [(-0.15) -(-0.14)] | -27.3 | <0.0001 | -0.1024 | [(-0.110)-( -0.0951)] |
| SPARE-Tau | Global 2 | -80.8 | <0.0001 | -0.1509 | [(-0.155)-( -0.147)] | -55.5 | <0.0001 | -0.2422 | [(-0.251)-( -0.234)] |
| Average Braak | Meta-Temporal ROI | 32.1 | <0.0001 | 0.056 | [(0.053)-( 0.059)] | -16.1 | <0.0001 | -0.0337 | [(-0.0378)-( -0.0296)] |
| Average Braak | CSF p-Tau | 149.1 | <0.0001 | 0.4435 | [(0.438)-( 0.449)] | 13.3 | <0.0001 | 0.0356 | [(0.0303)-( 0.0408)] |
| Average Braak | SPARE-AD | 20.7 | <0.0001 | 0.0521 | [(0.0471)-( 0.0570)] | -11.3 | <0.0001 | -0.0314 | [(-0.0368)-( -0.0259)] |
| Average Braak | Florbetapir Composite | 80.3 | <0.0001 | 0.254 | [(0.248)-( 0.260)] | -17.4 | <0.0001 | -0.0839 | [(-0.0934)-( -0.0744)] |
| Average Braak | Global 1 | -83.7 | <0.0001 | -0.1779 | [(-0.182)-( -0.174)] | -44.3 | <0.0001 | -0.1955 | [(-0.204)-( -0.187)] |
| Average Braak | Global 2 | -89.3 | <0.0001 | -0.1868 | [(-0.191)-( -0.183)] | -77.7 | <0.0001 | -0.3352 | [(-0.344)-( -0.327)] |
| Meta-Temporal ROI | CSF p-Tau | 129.8 | <0.0001 | 0.3875 | [(0.382)-( 0.393)] | 21.4 | <0.0001 | 0.0693 | [(0.0629)-( 0.0757)] |
| Meta-Temporal ROI | SPARE-AD | -1.4 | 0.16 | -0.0039 | [(-0.00934)-( 0.00153)] | 0.7 | 0.48 | 0.0024 | [(-0.0042)-( 0.0089)] |
| Meta-Temporal ROI | Florbetapir Composite | 58.1 | <0.0001 | 0.198 | [(0.191)-( 0.205)] | -9.4 | <0.0001 | -0.0502 | [(-0.0607)-( -0.0397)] |
| Meta-Temporal ROI | Global 1 | -98.6 | <0.0001 | -0.2339 | [(-0.239)-( -0.229)] | -34.6 | <0.0001 | -0.1618 | [(-0.171)-( -0.153)] |
| Meta-Temporal ROI | Global 2 | -105.7 | <0.0001 | -0.2427 | [(-0.247)-( -0.238)] | -64.7 | <0.0001 | -0.3015 | [(-0.311)-( -0.292)] |
| CSF p-Tau | SPARE-AD | -116.5 | <0.0001 | -0.3914 | [(-0.398)-( -0.384)] | -24 | <0.0001 | -0.0669 | [(-0.0724)-( -0.0615)] |
| CSF p-Tau | Florbetapir Composite | -50.9 | <0.0001 | -0.1895 | [(-0.197)-( -0.182)] | -26.8 | <0.0001 | -0.1195 | [(-0.128)-( -0.111)] |
| CSF p-Tau | Global 1 | -235.5 | <0.0001 | -0.6214 | [(-0.627)-( -0.616)] | -47.5 | <0.0001 | -0.2311 | [(-0.241)-( -0.222)] |
| CSF p-Tau | Global 2 | -249.6 | <0.0001 | -0.6303 | [(-0.635)-( -0.625)] | -80.2 | <0.0001 | -0.3708 | [(-0.380)-( -0.362)] |
| SPARE-AD | Florbetapir Composite | 58.2 | <0.0001 | 0.2019 | [(0.195)-( 0.209)] | -10.6 | <0.0001 | -0.0526 | [(-0.0622)-( -0.0429)] |
| SPARE-AD | Global 1 | -94 | <0.0001 | -0.23 | [(-0.235)-( -0.225)] | -36.1 | <0.0001 | -0.1641 | [(-0.173)-( -0.155)] |
| SPARE-AD | Global 2 | -99.5 | <0.0001 | -0.2388 | [(-0.244)-( -0.234)] | -68 | <0.0001 | -0.3039 | [(-0.313)-( -0.295)] |
| Florbetapir Composite | Global 1 | -130 | <0.0001 | -0.4319 | [(-0.438)-( -0.425)] | -17.5 | <0.0001 | -0.1116 | [(-0.124)-( -0.0991)] |
| Florbetapir Composite | Global 2 | -138.9 | <0.0001 | -0.4408 | [(-0.447)-( -0.435)] | -45.9 | <0.0001 | -0.2513 | [(-0.262)-( -0.241)] |
| Global 1 | Global 2 | -5.7 | <0.0001 | -0.0088 | [(-0.0119)-( -0.0058)] | -29.8 | <0.0001 | -0.1397 | [(-0.149)-( -0.131)] |
