# Supplementary material for: SPARE-Tau: A flortaucipir machine-learning derived early predictor of cognitive decline
Source: PLoS One. 2022 Nov 3;17(11):e0276392. doi: 10.1371/journal.pone.0276392 (PMC9632811; doi:10.1371/journal.pone.0276392)
Supplement: S4 Table — The top part of the table represents prediction based on cutoffs, and the bottom part evaluates z-scored biomarker values. (DOCX) [file pone.0276392.s005.docx]

**Supplementary Table 4. Association of baseline CSF p-tau and SPARE-Tau with longitudinal ADAS-Cog13 changes in the participant subset with CSF samples.** The top part of the table represents prediction based on cutoffs, and the bottom part evaluates z-scored biomarker values.

| Cutoff-Based Prediction | | | | | | | | | |
| --- | --- | --- | --- | --- | --- | --- | --- | --- | --- |
|  | All Participants | | | CU Aβ- | | | CU Aβ+ | | |
|  | Coef. (SE) | p-value | AIC | Coef. (SE) | p-value | AIC | Coef. (SE) | p-value | AIC |
| Flortaucipir SPARE-Tau index | 0.41 (0.06) | <0.0001 | 1995.6 | 0.44 (0.24) | 0.1 | 657.8 | 0.38 (0.1) | 0.0005 | 497.1 |
| CSF P-Tau | 0.12 (0.06) | 0.059 | 2068.3 | 0.02 (0.1) | 0.84 | 664.6 | 0.02 (0.11) | 0.84 | 512.9 |
| Z-Scored vlause-based Prediction | | | | | | | | | |
| Flortaucipir SPARE-Tau index | 0.06 (0.01) | <0.0001 | 1959.7 | 0.2 (0.06) | 0.0012 | 653.9 | 0.08 (0.02) | 0.0001 | 489.2 |
| CSF P-Tau | 0.04 (0.01) | 0.0042 | 2062.8 | 0.02 (0.03) | 0.61 | 667.6 | -0.01 (0.02) | 0.81 | 518.6 |

P-values corrected for multiple comparison using Benjamini-Hochberg Procedure.
